# Breaking the co-operation between bystander T-cells and natural killer cells prevents the development of immunosuppression after traumatic skeletal muscle injury in mice

**DOI:** 10.1042/CS20140835

**Published:** 2015-03-19

**Authors:** Florian Wirsdörfer, Jörg M. Bangen, Eva Pastille, Wiebke Hansen, Stefanie B. Flohé

**Affiliations:** *Surgical Research, Department of Trauma Surgery, University Hospital Essen, University Duisburg-Essen, 45147 Essen, Germany; †Department of Molecular Cell Biology, Institute of Cell Biology (Cancer Research), Medical Faculty, University Duisburg-Essen, 45147 Essen, Germany; ‡Institute of Medical Microbiology, University Hospital Essen, University Duisburg-Essen, 45147 Essen, Germany

**Keywords:** cytokines, immunosuppression, injury, myeloid differentiation factor 88 (MyD88), natural killer cells, regulatory T-cells, aa, amino acid, APC, antigen-presenting cell, BMDC, bone marrow–derived dendritic cell, CD, cluster of differentiation, CFSE, carboxyfluorescein succinimidyl ester, DAMP, damage-associated molecular pattern, DC, dendritic cell, FCS, fetal calf serum, FoxP3, transcription factor forkhead box P3, GM1, ganglioside monosialic acid, HMGB1, high mobility group box 1, HSP, heat shock protein, i.v., intravenous, IFN, interferon, IL, interleukin, MHC, major histocompatibility complex, MyD88, myeloid differentiation factor 88, NK, natural killer, OVA, ovalbumin, PAMP, pathogen-associated molecular pattern, pLN, popliteal lymph node, pOVA, OVA peptide, RAG, recombination-activating gene, s.c., subcutaneously, Th, T helper, Th1, Th type 1, TLRs, toll-like receptors, Tregs, regulatory T-cells, wt, wild-type

## Abstract

Nosocomial infections represent serious complications after traumatic or surgical injuries in intensive care units. The pathogenesis of the underlying immunosuppression is only incompletely understood. In the present study, we investigated whether injury interferes with the function of the adaptive immune system in particular with the differentiation of antigen-specific T helper (Th)-cell responses *in vivo*. We used a mouse model for traumatic gastrocnemius muscle injury. Ovalbumin (OVA), which served as a foreign model antigen, was injected into the hind footpads for determination of the differentiation of OVA-specific Th-cells in the draining popliteal lymph node (pLN). The release of interferon (IFN)-γ from OVA-specific Th-cells was impaired within 24 h after injury and this impairment persisted for at least 7 days. In contrast, the proliferation of OVA-specific Th-cells remained unaffected. Injury did not modulate the function of antigen-presenting cells (APCs) in the pLN. Adoptive transfer of total T-cells from pLNs of injured mice inhibited IFN-γ production by OVA-specific Th-cells in naive mice. Suppressed Th1 priming did not occur in lymphocyte-deficient mice after injury but was restored by administration of T-cells before injury. Moreover, the suppression of Th1 differentiation required the presence of natural killer (NK) cells that were recruited to the pLN after injury; this recruitment was dependent on lymphocytes, toll-like receptor 4 (TLR4) and myeloid differentiation factor 88 (MyD88). In summary, upon traumatic skeletal muscle injury T-cells and NK cells together prevent the development of protective Th1 immunity. Breaking this co-operation might be a novel approach to reduce the risk of infectious complications after injury.

## Abstract 2

After traumatic skeletal muscle injury, natural killer (NK) cells are recruited to the draining lymph node in a TLR4 (toll-like receptor 4)–myeloid differentiation factor 88 (MyD88)-dependent manner and impair the development of specific T-cell responses. The suppressed T-cell function might increase the susceptibility of severely injured patients to nosocomial infections.

## INTRODUCTION

Severely injured patients or patients undergoing major surgery experience inflammation-induced multiple organ failure but also are at high risk of nosocomial infections [1–3]. Multi-resistant bacteria are an increasing problem in healthcare worldwide and demand novel strategies to strengthen the patients' immune defence mechanisms. However, the origin of injury-induced immunosuppression is only poorly understood and is ascribed at least in part to impaired responsiveness of T-lymphocytes [4–6].

Upon injury-induced tissue destruction, endogenous molecules, such as heat shock proteins (HSPs), high-mobility group box 1 (HMGB1) or S100 proteins, are released from necrotic cells and trigger ‘sterile’ inflammation (reviewed in [7]). These molecules act as danger signals and are also called damage-associated molecular patterns (DAMPs) in analogy to pathogen-associated molecular patterns (PAMPs), such as lipopolysaccharide from Gram-negative bacteria. PAMPs and DAMPs share an overlapping set of recognition receptors, especially within the family of toll-like receptors (TLRs), which are largely expressed by innate immune cells [8].

To initiate a primary T-cell response, antigen-presenting cells (APCs) take up antigens, process them intracellularly and present antigen peptides in conjunction with major histocompatibility complex (MHC) class II molecules on the cell surface. Moreover, in the presence of PAMPs or DAMPs, APCs increase the expression of the co-stimulatory molecules cluster of differentiation (CD)40 and CD86 and release pro-inflammatory cytokines.

The immune response is further driven by the activation of CD4^+^ T helper (Th)-cells triggered by the interaction between specific receptors on the T-cells and antigen peptide-MHC class II and co-stimulatory molecules on the APCs. Activated Th-cells increase the expression of CD25 and CD69, proliferate and differentiate toward interferon (IFN)-γ-secreting Th type 1 (Th1)-cells; toward Th2-cells that release interleukin (IL)-4, IL-5 and IL-13; towards Th17-cells; or towards regulatory T-cells (Tregs) [9]. Tregs suppress the proliferation and cytokine response of effector T-cells and thereby control the extent of the immune response. Well-known Tregs are CD4^+^CD25^+^ T-cells that express the transcription factor forkhead box P3 (FoxP3) [10].

Among ‘professional’ APCs, dendritic cells (DCs) that reside in peripheral tissues and in lymphoid organs are the most potent because of their strong expression of MHC class II and co-stimulatory molecules and their release of the Th1-promoting cytokine IL-12 [11]. DCs take up foreign antigens in the periphery and migrate to draining lymph nodes, where they induce the antigen-specific Th-cell response [12]. Effective immune responses to pathogens often require the production of IFN-γ, which may be delivered by Th1-cells or by natural killer (NK) cells and which stimulates phagocytes to eliminate the infectious agent.

Diverse types of injury, such as haemorrhage, ischaemia/reperfusion, bone fracture and burns, are well-known inducers of remote organ damage due to sterile inflammation [13–15]. In contrast, little is known about the pathogenesis of immunosuppression, particularly after injuries induced by mechanical disruption of soft tissues, such as skeletal muscle injury, although this type of injury is inherently involved in both severe injury and major surgery [2,16,17].

In the present study, we investigated potential mechanisms that are involved in the development of immunosuppression after traumatic injury. We used a previously established mouse model of traumatic skeletal muscle injury [18]. Considering the evidence for disturbed T-cell function in severely injured patients we evaluated the impact of skeletal muscle injury on *in vivo* antigen-specific Th-cell priming.

## MATERIALS AND METHODS

### Mice

Male BALB/c mice, 10–12 weeks old, were obtained from Harlan Winkelmann. Male DO11.10, myeloid differentiation factor (MyD)88^−/−^, TLR4^−/−^ and recombination-activating gene (RAG) 2^−/−^ mice (all on BALB/c background) were bred at the local animal facilities of the University Hospital Essen and 10–12-week-old mice were used. DO11.10 mice carry a transgene for the αβ T-cell receptor of the amino acid (aa) sequence 323–339 of OVA. Mice were maintained under specific pathogen-free conditions and had access to standard rodent food and water *ad libitum*. All animal experiments were performed in accordance with the ethical principles and guidelines for scientific experiments on animals of the Swiss Academy of Medical Sciences and were approved by the local Landesamt für Natur, Umwelt und Verbraucherschutz (LANUV), North Rhine-Westphalia (Az. 9.93.2.10.34.07.288).

### Mechanical muscle injury

Mice were anaesthetized by an intramuscular (i.m.) injection of 58 mg/kg body weight ketamine and 7 mg/kg body weight xylazine (both from Ceva Sante Animale). Mechanical muscle injury was induced as previously described [18]. Briefly, a 20-g drop mass was dropped from a height of 1.20 m to deliver a single impact to the posterior part of the gastrocnemius muscle. Injury was induced on both hind limbs. Sham-treated animals were anaesthetized only. The induction of muscle injury neither damaged the lymphatic vessels nor fractured bones.

In some experiments, splenic CD3^+^ T-cells from wild-type (wt) mice were administered intravenously (i.v.) into RAG2^−/−^ mice 24 h before injury or sham treatment.

NK cells in spleens and lymph nodes were depleted by intraperitoneal (i.p.) application of 50 μl of anti-asialo ganglioside-monosialic acid (GM1) serum (Wako Chemicals) [19,20] 24 h before and every second day after injury or sham treatment. As control, animals received normal rabbit serum (provided by the local animal facility).

### Cell culture medium

Very low endotoxin medium [‘VLE’ Roswell Park Memorial Institute medium (RPMI) 1640] supplemented with 10% fetal calf serum (FCS), 10 mM HEPES, 2 mM glutamine (all from Biochrom), 0.06 mg/ml penicillin, 0.02 mg/ml gentamicin and 0.05 mM 2-mercaptoethanol (all from Sigma–Aldrich) was used as a culture medium. When lymph node cells were taken from mice that had received bone marrow-derived dendritic cells (BMDCs), the FCS in the culture medium was replaced by 1% normal mouse serum (PAA Laboratories GmbH).

### Generation and loading of BMDCs with OVA

BMDCs were generated by culturing bone marrow cells from naive mice with 20 ng/ml recombinant murine granulocyte macrophage colony-stimulating factor (rGM–CSF; R&D Systems), as previously described [21]. On day 7, non-adherent cells were harvested and incubated with 100 μg/ml OVA (ovalbumin) protein for 2 h. The cells were then washed with PBS.

### Isolation and labelling of T-cells

CD3^+^ T-cells from the spleens of naive DO11.10 or BALB/c mice or from popliteal lymph nodes (pLNs) of sham-treated and injured mice were purified by using the Pan T-Cell Isolation Kit (Miltenyi Biotec) and magnetic-activated cell sorting (MACS) according to the manufacturer's instructions (>95% purity as assessed by flow cytometry). DO11.10 T-cells were labelled with 1.5 μM carboxyfluorescein succinimidyl ester (CFSE; Molecular Probes) for 12 min at 37°C. After incubation for 30 min in culture medium, the cells were washed in PBS. The dilution of CFSE by 50% upon each cell division indicates the extent of proliferation.

### OVA-specific T-cell activation *in vivo* and *in vitro*

For *in vivo* T-cell activation, we made use of the adoptive transfer of T-cells from DO11.10 mice that are transgenic for an OVA peptide (pOVA)-specific T-cell receptor. This T-cell transfer increases the frequency of OVA-specific T-cells in wt mice to levels that allow their visualization *ex vivo*. Five million CFSE-labelled T-cells from DO11.10 mice were injected i.v. into recipient mice at different time points after sham treatment or injury as indicated in the Figure legends. In one distinct experimental approach, the CFSE-labelled T-cells were administered together with 1×10^6^ purified CD3^+^ T-cells isolated from pLN cells of sham-treated or injured mice. Twenty-four hours after the administration of T-cells from DO11.10 mice, either 30 μg of OVA protein (containing >100 endotoxin units per mg protein; Sigma–Aldrich) dissolved in 30 μl of PBS or 1×10^5^ OVA-loaded BMDCs were injected subcutaneously (s.c.) into each hind footpad. Three days later, pLN cells were prepared by disrupting the lymph nodes in culture medium with a 23G needle. The pLN cells were restimulated with 0.1 or 1.0 μg/ml pOVA (aa 323–339; AnaSpec] *in vitro*. All cultures were set up in triplicate with 2×10^5^ cells per well (96-well flat-bottom plates) at a total volume of 200 μl. After 3 days, the amount of IFN-γ, IL-10 and IL-2 in the supernatants was determined by ELISA (R&D Systems). For tracking the uptake of OVA, 30 μg of OVA protein FITC-labelled OVA protein (Molecular Probes) was administered into the hind footpads without previous application of OVA-specific T-cells. After 24 h, pLN cells were isolated.

For *in vitro* T-cell assays, spleen cells from naive mice were depleted from CD3^+^ cells by using mouse anti-CD3ε antibodies (clone 17A2; BD Biosciences) and sheep anti-rat IgG Dynabeads at a ratio of two beads per cell (Invitrogen) in combination with Dynal MPC-1 Magnetic Particle Concentrator (Dynal A.S.) and were used as APCs with a purity of more than 95% CD3^−^ cells. Next, 3×10^5^ APCs that had been irradiated with 30 Gy were co-cultured with 2×10^5^ CFSE-labelled T-cells from naive DO11.10 mice, along with CD3^+^ T-cells from pLNs of sham-treated or injured mice at various ratios. All cultures were set up in triplicate (96-well plates) in the presence or absence of 0.1 μg/ml pOVA. After 3 days, supernatants were harvested and analysed by IFN-γ ELISA. In both T-cell assays, the dilution of CFSE during the proliferation of OVA-specific T-cells was assessed by flow cytometry.

### Flow cytometry

We stained pLN cells with fluorochrome-labelled antibodies against the T-cell receptor specific for pOVA (clone KJ1-26; Caltag) in combination with anti-CD4 (clone RM4-5) or in combination with anti-CD69 (clone H1.2F3) and anti-CD25 (clone PC61.5). For tracking OVA–FITC, pLN cells were stained with anti-CD11c (clone N418) and anti-CD11b (clone M1/70). Antibodies against CD11c, CD40 (clone HM40-3) and CD86 (clone GL1) were used to analyse DC. For characterization of NK cells, pLN cells were stained against CD3ε clone 145-2C11), CD11b and CD49b (clone DX5). All antibodies were obtained from BD Biosciences. Intracellular staining of FoxP3 was performed with the FoxP3/Transcription Factor Staining Buffer Set from eBioscience according to the manufacturer's instructions. For all specific antibodies, appropriate isotype antibodies served as negative control. Flow cytometry was performed with a FACSCalibur flow cytometer (BD Biosciences) and CellQuest Pro software (BD Biosciences).

### Statistical analyses

Data are expressed as means ± S.D. of triplicate cultures, individual mice or multiple experiments. Statistically significant differences between two or more groups were detected with Student's *t*-test or with one-way ANOVA followed by the Bonferroni post-test. Statistical significance was set at the level of *P*<0.05. Prism 5.0 software (Graph Pad) was used for statistical analyses and preparation of graphs.

## RESULTS

### Skeletal muscle injury impairs the differentiation of Th1-cells

To verify the extent of muscle injury, we used confocal microscopy to examine sections of the gastrocnemius muscles after mechanically-induced injury and sham treatment. Sham-treated animals exhibited highly structured organization of muscle fibres, with tight contact between adjacent cells and the peripherally located nuclei; these are characteristics of skeletal muscle cells (Supplementary Figure S1). By 24 h after injury, the co-ordinated structure had disappeared, the muscle cells had become rounded and the cell contacts were largely loosened. Numerous leucocytes infiltrated the damaged area, not only between neighbouring muscle cells but also in the cavities that developed after destruction of tissue organization. The disruption of tissue organization was maintained at least until day 4 after injury. On day 7 after injury, the contacts between muscle cells had been restored and the cavities were closed, but infiltrating leucocytes were still present (Supplementary Figure S1).

**Figure 1:**
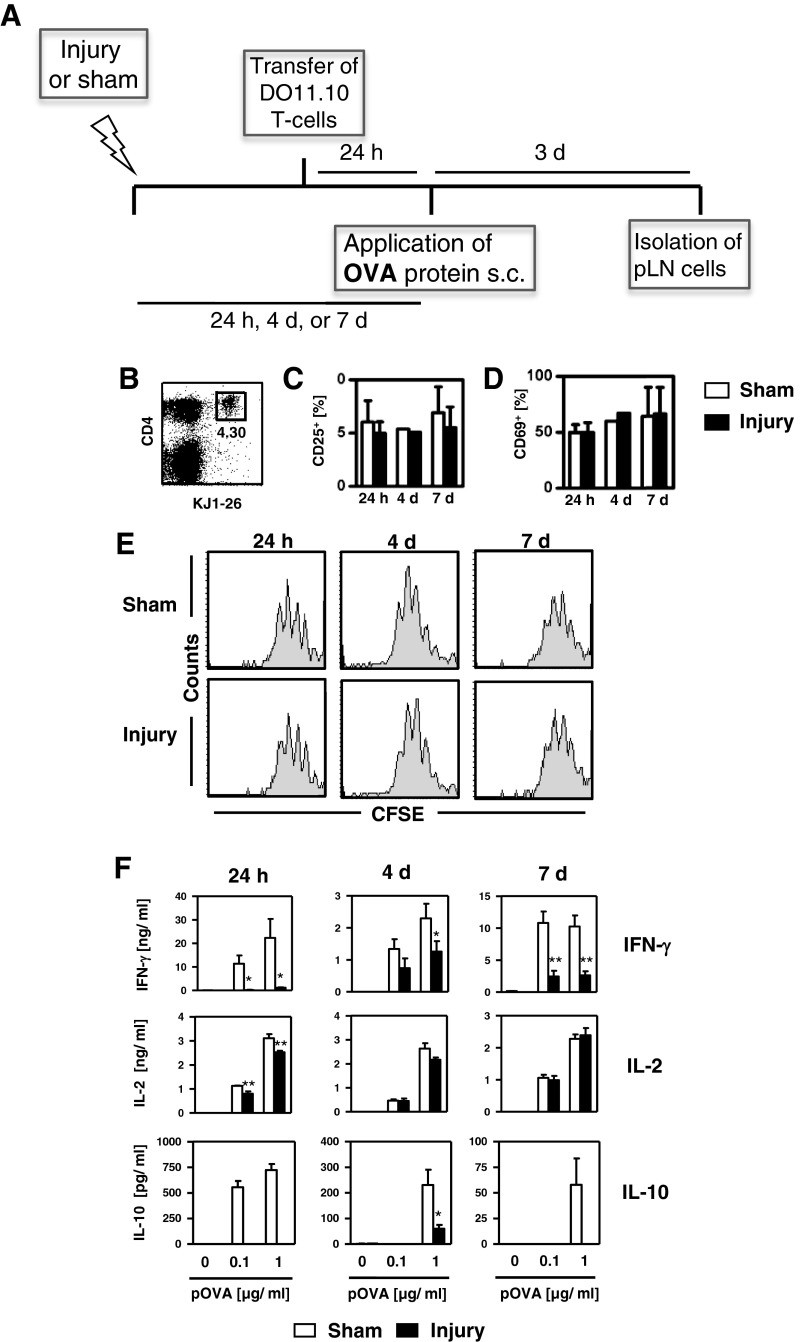
Traumatic skeletal muscle injury impairs the priming of Th1-cells in the draining lymph node By 24 h, 4 or 7 days after injury to the gastrocnemius muscle or after sham treatment, OVA was injected into the hind footpads of mice that had received CFSE-labelled T-cells from naive DO11.10 mice 24 h earlier. Three days after OVA administration, single-cell suspensions of pLNs were pooled per group and stained against CD4, KJ1-26, CD25 and CD69. (**A**) Overview of the experimental design. (**B**) Representative dot plot of pLN cells. OVA-specific Th-cells were identified as CD4^+^KJ1-26^+^ cells. The number indicates the percentage of OVA-specific Th-cells in the total population of pLN cells. Percentage of (**C**) CD25^+^ and (**D**) CD69^+^ cells among gated CD4^+^KJ1-26^+^ OVA-specific Th-cells. Bar graphs show mean ± S.D. of three experiments each with *n*=3 mice per group. (**E**) Histograms showing the proliferation of gated OVA-specific Th-cells according to the dilution of CFSE and are representative of three independent experiments. (**F**) pLN cells were restimulated with pOVA and the concentrations of IFN-γ, IL-2 and IL-10 in the supernatants were determined. Data show mean ± S.D. of triplicate cultures from pLN cells and are representative of three experiments each with *n*=3 mice per group. Statistical differences between sham-treated mice and injured mice were determined with Student's *t*-test. *P<0.05; **P<0.01.

We investigated whether this traumatic skeletal muscle injury affected the Th-cell response to a foreign antigen in draining pLNs. Preparations of OVA that contained endotoxin were used as a model antigen to mimic an infectious insult. OVA was injected into the hind footpads 24 h, 4 or 7 days after sham treatment or injury (for experimental design see Figure 1A). CFSE-labelled OVA-specific T-cells from naive DO11.10 mice were injected i.v. 24 h before the injection of OVA. After 3 days, pLN cells were isolated and OVA-specific Th-cells were identified as CD4^+^KJ1-26^+^ cells (Figure 1B). OVA-specific Th-cells from sham-treated and injured mice did not differ in their expression of the activation markers CD25 (Figure 1C) and CD69 (Figure 1D). Likewise, OVA-specific Th-cells proliferated similarly in the pLNs of sham-treated and injured mice (Figure 1E). The percentage of OVA-specific T-cells in total lymph node cells did not differ between the two groups (result not shown).

Upon restimulation of OVA-specific Th-cells with pOVA *in vitro*, pLN cells from both groups released comparable amounts of IL-2, although the injured mice exhibited a slight but significant reduction in IL-2 release 24 h after injury (Figure 1F). In contrast, at all three time points restimulated pLN cells from injured mice released lower levels of IFN-γ and IL-10 than did cells from sham-treated mice (Figure 1F). The concentration of the Th2 cytokines IL-4, IL-5 and IL-13 in the supernatants of cells from both groups was below the detection limit (result not shown). Lymph node cells from mice that had not received OVA-specific T-cells before the application of OVA did not secrete any of these Th1 or Th2 cytokines (result not shown). Thus, traumatic skeletal muscle injury interferes with the differentiation of Th1-cells in draining pLNs for at least 7 days.

### Transport of the antigen into the pLN

**Figure 2:**
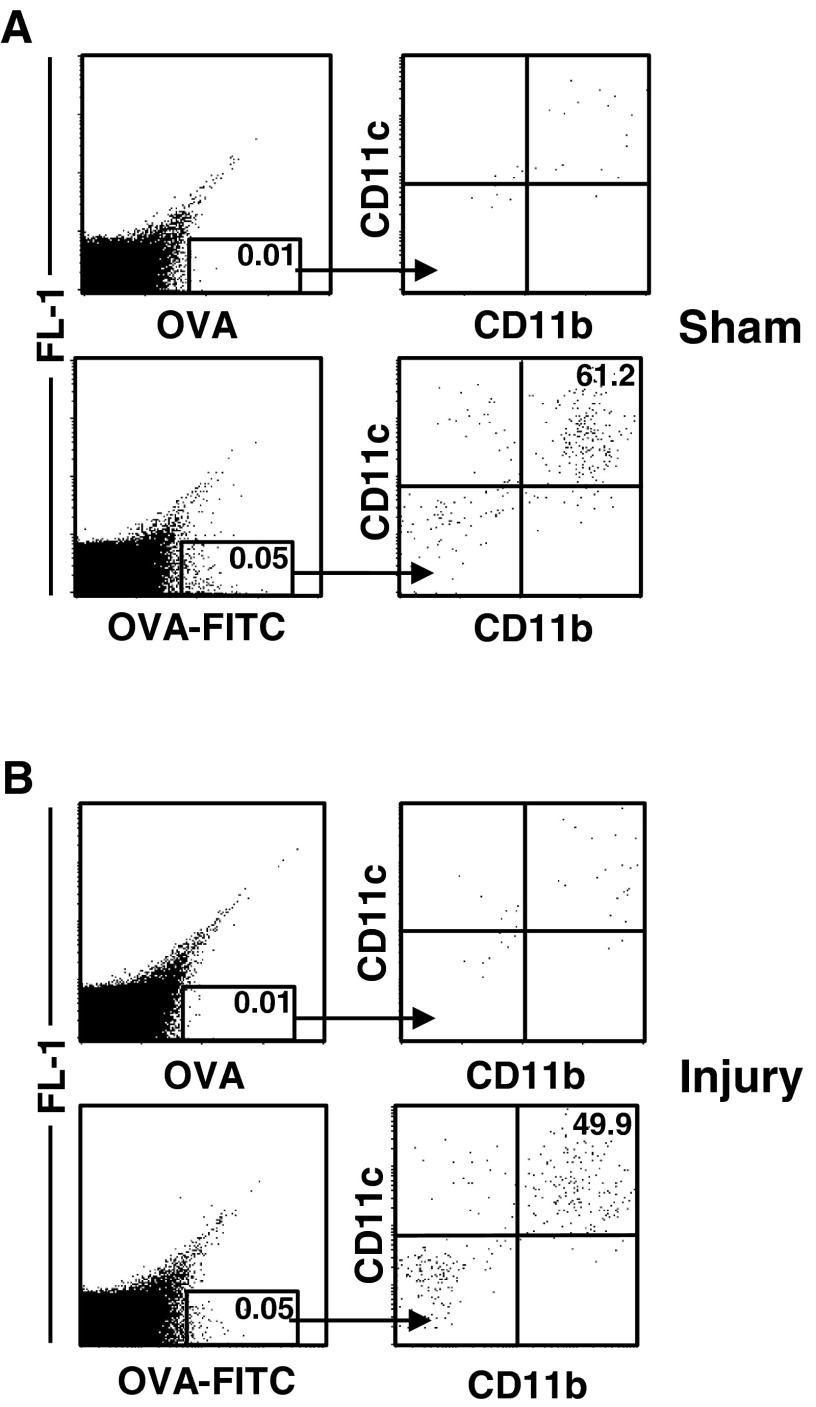
After injection into the footpads, OVA is internalized by DCs in the pLNs Seven days after injury or sham treatment, unlabelled OVA protein or OVA–FITC was injected into the hind footpads. After 24 h, pLN cells were pooled per group (*n*=3 mice each). OVA–FITC^+^ cells from (**A**) sham-treated mice and (**B**) injured mice were gated and analysed in terms of CD11b and CD11c expression. The threshold for OVA–FITC^+^ cells was set by using pLN cells from mice that had received unlabelled OVA. The numbers indicate the percentage of OVA–FITC^+^ cells among total pLN cells (left column) and of CD11c^+^CD11b^+^ cells among gated OVA–FITC^+^ cells (right column).

To address the potential mechanisms underlying altered T-cell differentiation after injury, we first investigated the transport of injected OVA to the draining lymph node, where T-cell priming takes place. To do so, we injected FITC-labelled OVA into the footpads 7 days after injury. Analysis of the pLN cells showed that by 24 h after the application of OVA, a population of CD11b^+^CD11c^+^OVA–FITC^+^ DCs appeared in the pLNs of both sham-treated (Figure 2A) and injured mice (Figure 2B). Thus, after traumatic skeletal muscle injury the lymphatic vessels remain intact and allow the transport of OVA into the draining lymph node.

### The T-cell stimulatory capability of pLN-resident APCs is not changed after injury

**Figure 3:**
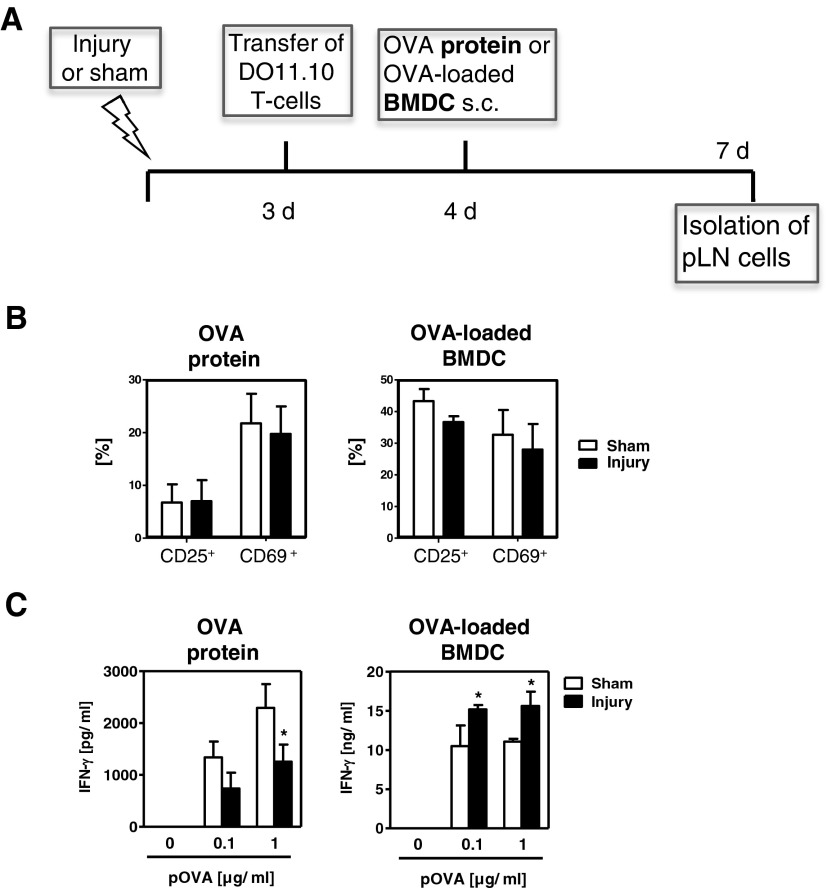
Inverse capability of exogenously loaded DCs and endogenous APCs to Th1-cell priming *in vivo* OVA protein or BMDCs previously loaded with OVA *in vitro* were injected into the hind footpads 4 days after injury or sham treatment and after transfer of CFSE-labelled OVA-specific Th-cells. After 3 days, pLN cells were pooled per group. (**A**) Overview of the experimental design. (**B**) Percentage of CD25^+^ and CD69^+^ cells in gated OVA-specific Th-cells. (**C**) Content of IFN-γ in the supernatants after restimulation of pLN cells with pOVA. Data show mean ± S.D. of triplicate cultures and are representative of three independent experiments with *n*=3 mice per group. Statistically significant differences were detected with Student's *t*-test. *P<0.05, sham treatment compared with injury.

Appropriate T-cell priming depends on the function of APCs, which may be altered after injury. To investigate the OVA-specific T-cell response independent of endogenous APC in the injured host, we loaded BMDCs from naive mice with OVA *in vitro*; these cells served as APCs *in vivo*. Four days after injury or sham treatment, mice received s.c. injections of OVA protein or naive OVA-loaded BMDCs into the hind footpads. CFSE-labelled OVA-specific T-cells had been transferred 24 h earlier (for experimental approach see Figure 3A). After 3 days, pLN cells were analysed in terms of OVA-specific T-cell proliferation and IFN-γ secretion *in vitro*, as described above. OVA-specific T-cells proliferated similarly in sham-treated and injured mice after the application of OVA protein and after the application of BMDCs. In general, T-cell proliferation was higher after the application of OVA protein and this higher proliferation contributed to a higher percentage of OVA-specific T-cells in the pLNs (result not shown). We observed no difference between OVA-specific T-cells from sham-treated and injured mice in their percentage in total lymph node cells (result not shown) and in their expression of CD25 and CD69 after the injection of either OVA protein or BMDCs (Figure 3B). As described above (Figure 1F), pLN cells from injured mice that had received OVA protein secreted less IFN-γ than pLNs from sham-treated mice after restimulation *in vitro* (Figure 3C). However, after the application of OVA-loaded BMDCs, pLN cells from injured mice released larger amounts of IFN-γ than did cells from sham-treated mice (Figure 3C).

The absence of impaired IFN-γ secretion from OVA-specific T-cells in injured mice upon the application of antigen-loaded BMDCs pointed to the involvement of endogenous APCs in T-cell suppression after injury. To address this issue, we studied the expression of co-stimulatory molecules and the T-cell stimulatory capability of APCs. The percentage of CD11c^+^ DCs in the pLNs of sham-treated and injured mice did not differ (Supplementary Figure S2A), nor did their expression of CD40 and CD86 (Supplementary Figure S2B). Accordingly, isolated APCs from pLNs of sham-treated and injured mice exhibited comparable ability to induce OVA-specific Th-cell proliferation (Supplementary Figure S2C) and IFN-γ secretion *in vitro* (Supplementary Figure S2D). Thus, after skeletal muscle injury, the priming of antigen-specific Th-cells by resident APCs in the pLNs is not restricted and the Th1 differentiation ability of transferred OVA-specific T-cells is not generally impaired.

### Endogenous T-cells mediate the suppression of OVA-specific Th-cells after injury

**Figure 4:**
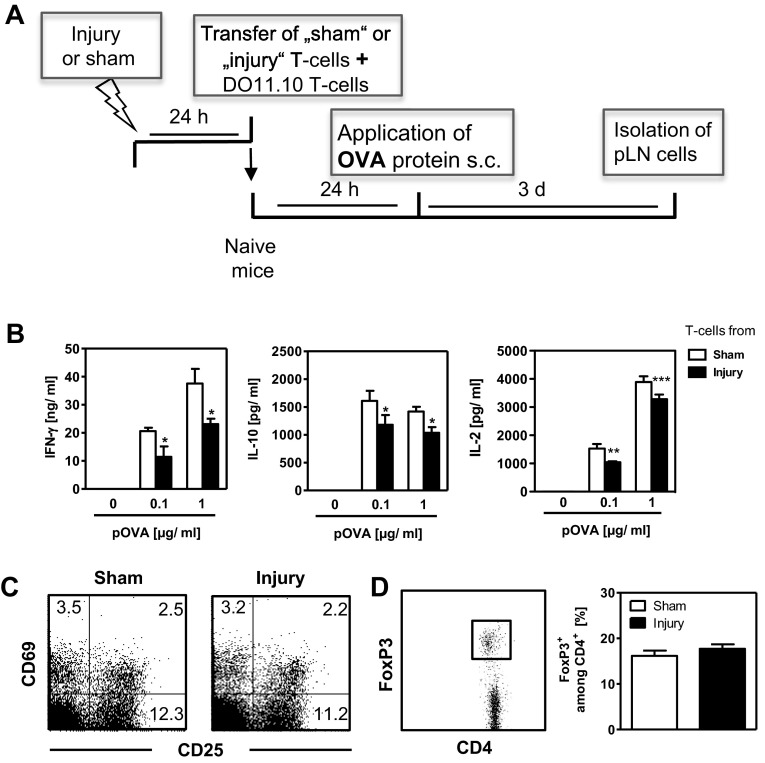
Regulatory activity of endogenous T-cells from pLNs after injury *in vivo* Twenty-four hours after injury or sham treatment, CD3^+^ T-cells were isolated from pLNs and were adoptively transferred into naive mice together with CFSE-labelled OVA-specific T-cells from DO11.10 mice. OVA was injected into the hind footpads and pLN cells were isolated and pooled per group 3 days later. (**A**) Overview of the experimental design. (**B**) Content of IFN-γ, IL-10 and IL-2 in the supernatants of pLN cells after restimulation with pOVA. Data show mean ± S.D. of triplicate cultures from pLN cells and are representative of three independent experiments (*n*=3 mice each). Statistically significant differences between sham-treated mice and injured mice were detected with Student's *t*-test. *P<0.05; **P<0.01; ***P<0.001. (**C** and **D**) The pLN cells were isolated 24 h after injury or sham treatment (no transfer of OVA-specific T-cells). (**C**) Dot plots show the expression of CD25 and CD69 by gated CD4^+^ T-cells. The numbers indicate the percentage within the respective quadrants. (**D**) FoxP3 expression in gated CD4^+^ T-cells. Bar graph shows the mean percentage of FoxP3^+^ among CD4^+^ Th-cells from pLN cells of individual mice (*n*=3 mice each). Data are representative of two experiments.

Tregs are frequently involved in Th-cell suppression. We investigated whether endogenous T-cells acquire regulatory activity after injury and suppress the priming of subsequently transferred OVA-specific Th-cells. Therefore, we isolated CD3^+^ T-cells from pLNs 24 h after injury or sham treatment and transferred them into naive mice, along with naive CFSE-labelled OVA-specific T-cells. One day later, we injected OVA s.c. into the footpads and 3 days later we measured the proliferation, activation and cytokine secretion of OVA-specific T-cells from pLN cells (for experimental approach see Figure 4A). Irrespective of whether the T-cells injected into the naive mice came from sham-treated or injured mice, the co-injected OVA-specific T-cells proliferated to the same extent and reached the same percentages among total lymph node cells (result not shown). In contrast, the transfer of T-cells from injured mice induced a clearly reduced OVA-specific release of IFN-γ from pLN cells after restimulation *in vitro*, as well as a slightly diminished production of IL-10 and IL-2 (Figure 4B). Analysis of the T-cells from pLNs of sham-treated and injured mice that were used for adoptive transfer into naive mice found no difference in the expression of either CD25 or CD69 (Figure 4C) or in the percentage of CD4^+^FoxP3^+^ Tregs (Figure 4D).

**Figure 5:**
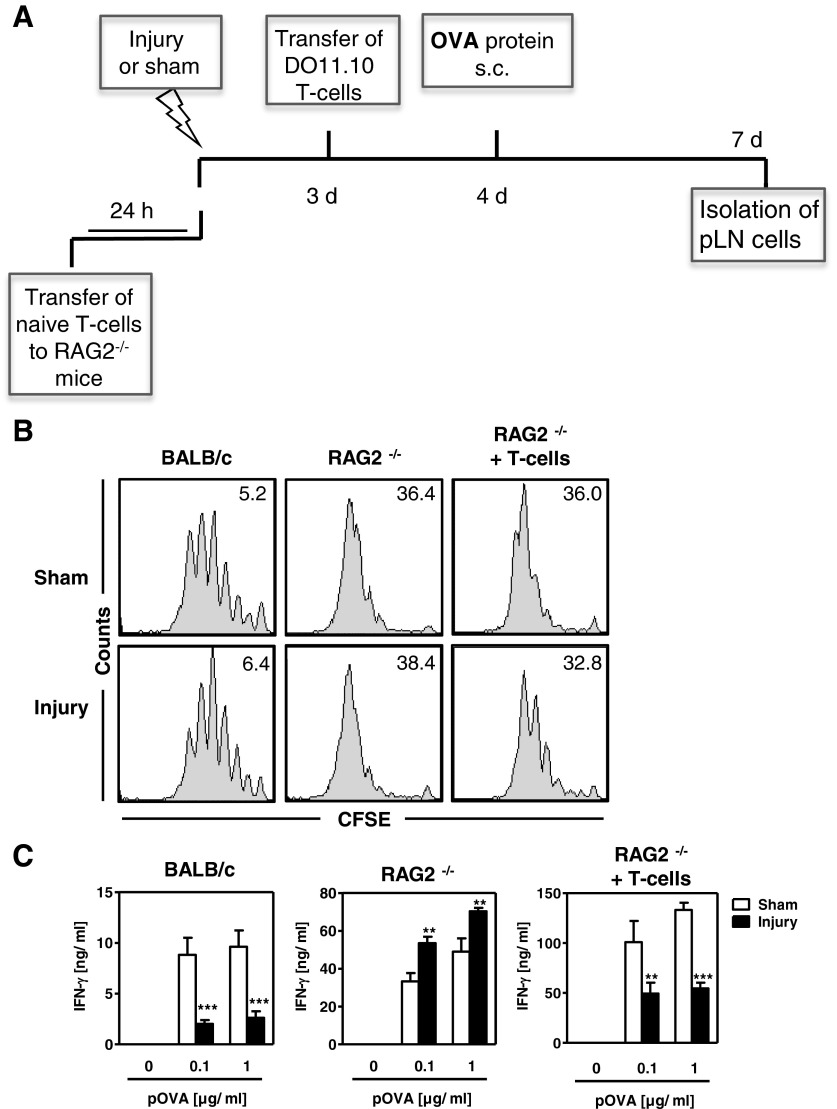
Role of endogenous T-cells in injury-induced Th1-cell suppression Wt BALB/c mice RAG2^−/−^ mice or RAG2^−/−^ mice that had received purified CD3^+^ T-cells from naive BALB/c mice 24 h earlier underwent injury or sham treatment. CFSE-labelled T-cells from DO11.10 mice were applied, OVA protein was injected into the footpads and pLN cells were pooled per group (*n*=3 mice each). (**A**) Overview of the experimental design. (**B**) Representative histograms of CFSE dilution in gated OVA-specific Th-cells of pLN cells. (**C**) The pLN cells were restimulated with pOVA and the concentration of IFN-γ was determined. Data show mean ± S.D. of triplicate cultures (*n*=3 mice per group) and are representative of three independent experiments. Statistically significant differences between sham-treated mice and injured mice were detected with Student's *t*-test. **P<0.01; ***P<0.001.

To further investigate the relevance of endogenous or ‘bystander’ T-cells in the development of OVA-specific T-cell suppression after injury, we examined the OVA-specific Th-cell response in RAG2^−/−^ mice that lack mature T- and B-lymphocytes. Sham treatment or injury was applied to wt BALB/c mice, to RAG2^−/−^ mice or to RAG2^−/−^ mice that had been reconstituted with CD3^+^ T-cells from naive wt mice 24 h earlier. The OVA-specific Th-cell response was investigated, as described above, by transferring CFSE-labelled OVA-specific T-cells and applying OVA protein (for experimental design, see Figure 5A). In each experimental setting, the proliferation of CD4^+^KJ1-26^+^ OVA-specific T-cells did not differ between pLN cells from injured mice or from sham-treated mice (Figure 5B). Because of the homoeostatic proliferation of T-cells that occurs after transfer into RAG2^−/−^ mice [22], the proliferation and the percentage of transferred OVA-specific T-cells was generally higher in RAG2^−/−^ mice than in wt mice (Fig. 5B). Accordingly, after restimulation with pOVA *in vitro*, higher levels of IFN-γ were released by pLN cells from RAG2^−/−^ mice than by pLN cells from wt mice. In contrast to pLN cells from wt mice, pLN cells from injured RAG2^−/−^ mice secreted higher amounts of IFN-γ than did pLN cells from sham-treated RAG2^−/−^ mice (Figure 5C). When RAG2^−/−^ mice were reconstituted with naive T-cells before injury or sham treatment, the secretion of IFN-γ by restimulated pLN cells from injured mice was impaired and was similar to that of pLN cells from wt mice (Figure 5C).

**Figure 6:**
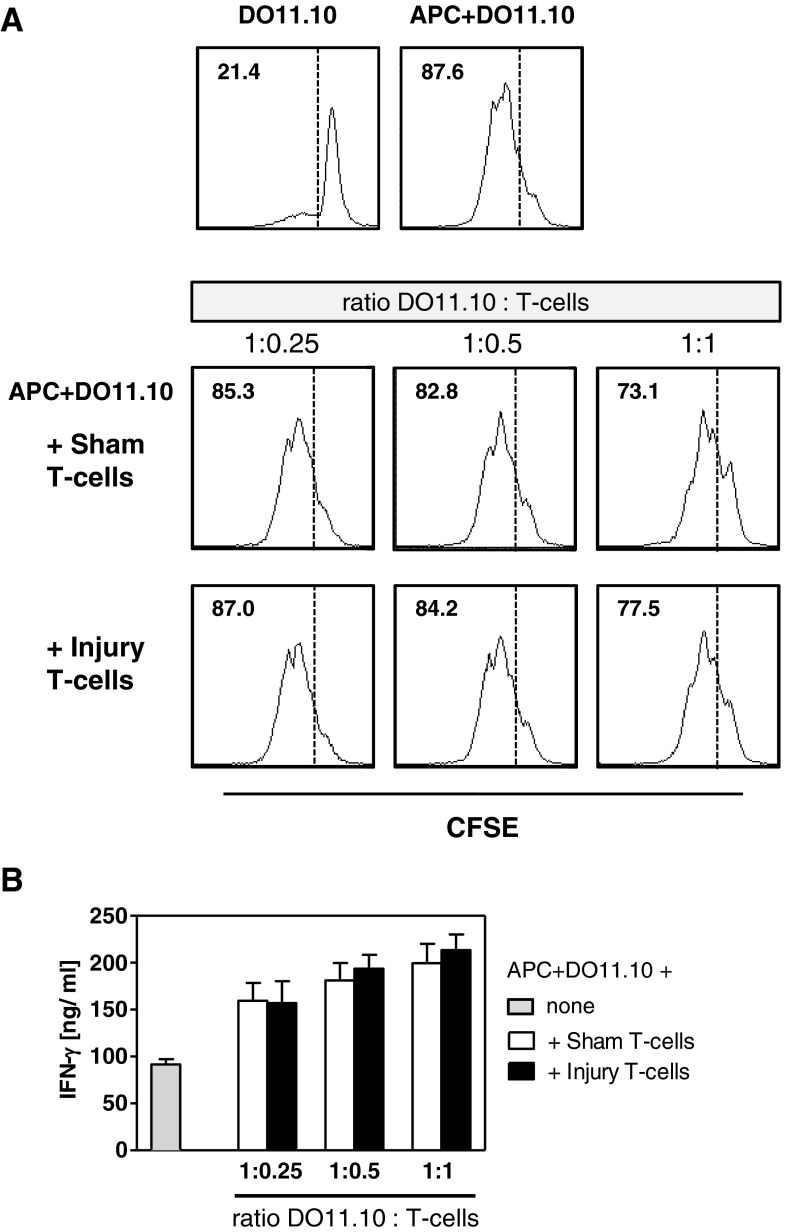
Endogenous T-cells from injured mice possess no regulatory activity *in vitro* APCs from naive mice were co-cultured with CFSE-labelled CD3^+^ T-cells from DO11.10 mice in the presence of pOVA. Pooled CD3^+^ T-cells from pLNs isolated 24 h after injury or sham treatment (*n*=3 mice per group) were added to the co-cultures at various ratios, as indicated. CFSE-labelled CD3^+^ T-cells alone served as a negative control. (**A**) Representative histograms of CFSE dilution after 3 days of culture. The numbers indicate the percentage of proliferating DO11.10 cells. Dashed lines separate proliferating and non-proliferating cells. (**B**) Concentration of IFN-γ in the supernatants. Data show mean ± S.D. of triplicate cultures. Data on proliferation and IFN-γ secretion are representative of five independent experiments.

Next, we performed additional *in vitro* analyses of the ability of endogenous T-cells from injured and sham-treated mice to inhibit OVA-specific T-cells. Therefore, irradiated APCs from naive mice and CFSE-labelled OVA-specific T-cells were cultured with pOVA in the presence of titrated numbers of T-cells obtained from the pLNs of mice 24 h after injury or sham treatment. The proliferation of OVA-specific T-cells and the content of IFN-γ in the supernatants were determined. In the presence of pOVA-loaded APCs, OVA-specific T-cells strongly proliferated and released IFN-γ (Figures 6A and 6B). The addition of T-cells from sham-treated or injured mice slightly reduced the proliferation of OVA-specific T-cells (Figure 6A) but increased their release of IFN-γ (Figure 6B) to a similar extent. Thus, endogenous T-cells from injured mice suppress the priming of antigen-specific Th-cells *in vivo* but not *in vitro*.

### NK cells contribute to the development of Th-cell suppression after injury

**Figure 7:**
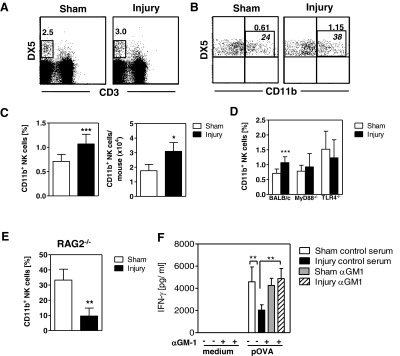
NK cells mediate the development of Th-cell suppression after injury (**A—E**) Twenty-four hours after injury or sham treatment of BALB/c mice and of MyD88^−/−^, TLR4^−/−^ and RAG2^−/−^ mice, pLN cells were analysed for the presence of CD3^−^DX5^+^ NK cells and their expression of CD11b. (**A**) Gating strategy of total CD3^−^DX5^+^ NK cells. The numbers indicate the percentage of NK cells among total pLN cells. (**B**) Dot plots showing the expression of CD11b on NK cells. The numbers indicate the percentage of CD11b^+^ NK cells among total pLN cells (upper line) and among total NK cells (lower line). Dot plots are representative of three experiments with *n*=4 mice each. (**C**) Percentage of CD11b^+^ NK cells among total pLN cells and absolute numbers of CD11b^+^ NK cells per mouse (BALB/c). Data show mean ± S.D. of individual mice from one of the three experiments with *n*=4 mice each. (**D**) Percentage of CD11b^+^ NK cells in pLN cells from BALB/c mice (already shown in **C**) in comparison with pLN cells from MyD88^−/−^ and TLR4^−/−^ mice. (**E**) Percentage of CD11b^+^ NK cells in pLN cells from RAG2^−/−^ mice. Statistically significant differences were detected with Student's *t*-test. (**F**) BALB/c mice were treated with an anti-asialo antiserum (αGM-1) or control serum before and after injury or sham treatment as described in the section ‘Materials and Methods’. OVA-specific T-cells were applied, OVA was injected into the hind footpads and pLN cells were pooled per group (*n*=3 mice each). The release of IFN-γ from restimulated pLN cells was determined. Data show mean ± S.D. of triplicate cultures and are representative of two experiments. Statistical differences were tested with one-way ANOVA followed by the Bonferroni post-test. *P<0.05; **P<0.01; ***P<0.001.

The absence of the ability of endogenous T-cells from injured mice to inhibit OVA-specific T-cells *in vitro* led to the assumption that an additional cell population might be required to accomplish antigen-specific T-cell suppression after injury, as observed *in vivo*. Analysis of the cellular composition of pLN cells found an increased percentage (Figures 7A and 7B) and a higher absolute number of mature CD3^−^DX5^+^CD11b^+^ NK cells (Figure 7C) 24 h after injury [23]. Because TLR signalling is involved in the activation and migration of NK cells during infection [24], we used MyD88^−/−^ and TLR4^−/−^ mice to assess the role of TLR signalling pathways in the recruitment of NK cells in our sterile injury model. No increase in the percentage of CD11b^+^ NK cells in the pLNs 24 h after injury was observed in MyD88^−/−^ or TLR4^−/−^ mice (Figure 7D). Considering the absence of the suppression of OVA-specific Th1-cell differentiation in injured RAG2^−/−^ mice, we examined the NK cell population in RAG2^−/−^ mice. Because of their lack of T- and B-lymphocytes, the pLNs of RAG2^−/−^ mice contained a high percentage of CD11b^+^ NK cells (Figure 7E). In contrast to pLNs from wt mice, pLNs from RAG2^−/−^ mice exhibited a strongly reduced percentage of CD11b^+^ NK cells after skeletal muscle injury (Figure 7E); this reduction was not associated with an increase in other myeloid cell populations (Supplementary Figure S3).

We further investigated the potential relevance of NK cells in the suppression of OVA-specific Th1-cell differentiation after injury. Upon administration of OVA into the footpads of sham-treated and injured mice, the population of CD11b^+^ NK cells in the pLNs of sham-treated and injured mice increased strongly and reached similar numbers (Supplementary Figure S4). In addition, we depleted NK cells in wt mice before sham treatment or injury by using an anti-asialo GM1 antiserum. Normal rabbit serum was used as a control. OVA-specific DO11.10 T-cells were transferred, OVA was injected into the hind footpads 4 days after injury or sham treatment and pLN cells were restimulated *in vitro* 3 days later. The depletion of NK cells had no effect on the proliferation or on the percentage of OVA-specific T-cells in either group (data not shown). The depletion of NK cells exerted no significant effect on the release of IFN-γ by restimulated pLN cells from sham-treated mice (Figure 7F). However, the depletion of NK cells before injury contributed to an OVA-specific increase in IFN-γ secretion by pLN cells up to the levels of IFN-γ released by pLN cells from sham-treated mice (Figure 7F). Thus, NK cells appear in draining lymph nodes after injury and contribute to the suppression of differentiation of antigen-specific Th1 cells.

## DISCUSSION

During the past three decades, various studies contributed to the evidence indicating that diverse types of injury interfere with the function of cells in the adaptive immune system, namely, the cytokine secretion pattern and proliferation of T-lymphocytes [25–28]. Frequently, such studies were based on *ex vivo* stimulation of total leucocytes with mitogens, such as phorbol-myristate acetate or concanavalin A, which do not necessarily reflect the responsiveness of T-cells upon recognition of their specific antigen *in vivo*. In the present study, we contributed to the knowledge about injury-induced T-cell modulation by *in vivo* tracking of antigen-specific Th-cells in the injured host after exposure to their cognate antigen.

We provide first evidence that traumatic skeletal muscle injury is sufficient to suppress the release of IFN-γ and IL-10 from OVA-specific Th-cells after their activation *in vivo*. In contrast, the antigen-specific proliferation of these cells and their release of IL-2 remain unaffected. Because IFN-γ is the characteristic cytokine of Th1-cells, we assume that skeletal muscle injury interferes with the polarization of Th-cells toward Th1. Unlike earlier reports on mitogen-induced T-cell activation after severe injury [28,29], our study did not find that the secretion of the Th2 cytokines IL-4, IL-5 or IL-13 is enhanced at any time point after injury (result not shown). We propose that the reduced polarization of antigen-specific T-cells towards Th1 after skeletal muscle injury does not occur in favour of the differentiation towards Th2.

IL-10 is a regulatory cytokine that is frequently associated with reduced secretion of Th1-type IFN-γ [30]. Therefore, our finding that the release of both IFN-γ and IL-10 is suppressed after the restimulation of OVA-specific Th-cells after injury was unexpected. Previous reports have described specific Th1-cells that release IFN-γ and IL-10 and differentiate under certain conditions, e.g. in the presence of high antigen concentrations [31]. The differentiation of such IFN-γ^+^IL-10^+^ OVA-specific Th1-cells in sham-treated animals but not in injured mice may explain the reduced OVA-specific production of both cytokines after injury, but this finding remains to be confirmed by further investigations.

Severe tissue injury rapidly causes inflammation and results in life-threatening damage to remote organs [32]. The concomitant suppression of Th1 priming after skeletal muscle injury may be an endogenous mechanism that aims to compensate for such overwhelming injury-induced inflammation. On the other hand, the inhibition of Th1 polarization is maintained for at least 7 days after injury. During this time frame, the immune response against invading pathogens may be disturbed; for example, the eradication of *Staphylococcus aureus* that requires the appropriate production of IFN-γ [33,34]. We propose that the damage to skeletal muscle tissue, a frequent element of accidental and surgical injury, favours the development of immunosuppression and contributes to the enhanced susceptibility of severely injured patients to infectious complications. Impaired eradication of *S. aureus* at the site of injury has been described after skeletal muscle injury in rats [35]; this finding supports our hypothesis about the association of muscle injury with the development of immunosuppression.

The extent of T-cell activation and subsequent T-cell polarization depends on multiple factors, such as antigen availability, APC-derived co-stimulatory signals and the cytokine micro-environment. The finding that the suppression of OVA-specific Th1-cell differentiation after injury does not occur when OVA-loaded BMDCs instead of endogenous cells serve as APCs implies that antigen presentation in the pLNs differs between sham-treated and injured mice and that OVA-specific T-cells are not generally suppressed after transfer into the injured host. Failure of appropriate APC function by DCs has previously been reported in murine cytomegalovirus infection and polymicrobial sepsis; this failure is associated with impaired IL-12 production, disturbance of co-stimulation and impaired ability to polarize toward Th1 [36–38]. Interestingly, we found no evidence that skeletal muscle injury interferes with the transport of OVA into the lymph nodes, with the expression of co-stimulatory molecules on DCs or with the T-cell stimulatory ability of APCs from draining lymph nodes. Therefore, the suppression of antigen-specific Th1 polarization after skeletal muscle injury does not seem to rely on a disturbance of APC function induced by tissue damage.

T-cell responses not only depend on APC but also are under the control of Tregs that limit the proliferation and cytokine secretion of effector T-cells. Our observation that CD3^+^ T-cells from injured mice transfer the suppression of antigen-specific Th1 polarization to naive mice implies that Tregs are involved in injury-induced inhibition of antigen-specific Th1-cell differentiation. This assumption was supported by the finding that the suppression of Th1 polarization after injury does not occur in RAG2^−/−^ mice that lack mature T- and B-lymphocytes but is restored by the transfer of CD3^+^ T-cells from naïve mice before injury. Well known T-cells with regulatory activity are Tregs that express the transcription factor FoxP3 and impair Th-cell effector function and proliferation by releasing IL-10 and transforming growth factor (TGF)-β [39]. The unchanged percentage of FoxP3^+^ Tregs in the lymph nodes after skeletal muscle injury indicates that the inhibition of OVA-specific Th1 polarization after muscle injury is not mediated by an expansion of the Treg population. However, since the number of Tregs in the pLNs were too small to allow us to perform functional assays with isolated Tregs, we cannot exclude the possibility that Tregs, although their percentage does not change, possess enhanced activity after skeletal muscle injury, like the Tregs in severely injured patients [40]. Thus, additional studies are needed to finally exclude the potential contribution of FoxP3^+^ Tregs to the suppression of Th1 polarization after skeletal muscle injury.

Purified endogenous T-cells from lymph nodes of injured mice transfer the suppression of OVA-specific Th1 polarization *in vivo* but do not exhibit the characteristic features of Tregs *in vitro*: inhibition of proliferation and IFN-γ secretion by antigen-specific effector T-cells [39]. This observation argues against the involvement of any type of T-cells with regulatory activity, including not only FoxP3^+^ Tregs but also FoxP3^−^ Tr1-cells and γδ T-cells that display features similar to those of FoxP3^+^ Tregs [10]. Therefore, we hypothesize that endogenous T-cells are necessary but are not sufficient for suppressing the Th1 polarization of transferred OVA-specific Th-cells after skeletal muscle injury.

NK cells not only possess cytotoxic activity but may also influence antigen-specific Th-cell differentiation in lymph nodes [41] by releasing IFN-γ. Therefore, we considered NK cells as a putative candidate for investigating injury-induced suppression of Th1-cell polarization. After an infectious insult, NK cells are recruited to the lymph node draining the site of infection [24,42]. We show in the present study for the first time that a traumatic skeletal muscle injury in the absence of an infectious agent is sufficient to induce the recruitment of CD11b^+^ NK cells to the lymph node draining the injured muscle. The injury-induced recruitment of NK cells is dependent on TLR4 and on the adapter protein MyD88. Diverse DAMPs are known to be released after injury and to signal, in part, by TLR4/MyD88; some of these DAMPs are HSP60, HMGB1 and distinct S100 proteins [32,43]. Such potential TLR4 agonists may stimulate APCs in the lymph nodes to release mediators that attract NK cells or they may act directly on circulating NK cells to promote their settling in the lymph nodes [24,42,44]. The identification of the putative TLR4 agonist and its cellular target is currently under investigation. Our findings show that, after the injection of OVA, numerous NK cells are recruited to the lymph node of sham-treated and injured mice and both populations are of similar size. Therefore, we assume that the suppressed OVA-specific Th1 polarization after injury does not rely on an inadequate number of NK cells during Th-cell priming.

In the absence of T-cells, NK cells fail to accumulate in the lymph node after skeletal muscle injury. This finding is in line with that of a previous report, which showed that T-cells mediate the mobilization of NK cells to the circulation and their recruitment to the site of inflammation [45]. Moreover, the depletion of NK cells before injury rescues Th1 priming in injured mice, a finding indicating that NK cells contribute to the suppression of Th1-cell differentiation after injury. NK cells that inhibit Th1 priming have been described and may act through soluble mediators such as IL-10 [46,47]. Very recently, IL-10-producing NK cells have been found in the spleen after haemorrhagic shock and are associated with impaired immune defence against opportunistic infections [48]. Trauma-haemorrhage and skeletal muscle injury differ in several aspects, e.g. in the origin of tissue damage (inflammation-induced injury after haemorrhage compared with mechanical disruption in the case of skeletal muscle injury). Therefore, the generation of regulatory NK cells may represent a common mechanism in the development of immunosuppression after injury. This assumption is further supported by our own study that shows that circulating NK cells from severely injured patients are inhibited in IFN-γ secretion in response to *S. aureus* (manuscript in preparation). The existence and function of such regulatory NK cells after traumatic injury is currently under investigation. We propose that endogenous or ‘bystander’ T-cells mediate the recruitment of NK cells into the lymph node after skeletal muscle injury and that both cell types co-operate in a so far unknown manner to prevent the development of an antigen-specific Th1 response.

In summary, we show in the present study for the first time that traumatic skeletal muscle injury triggers the recruitment of NK cells to the draining lymph node in a lymphocyte and TLR4–MyD88-dependent manner. The interaction of NK cells and endogenous T-cells is associated with the development of immunosuppression indicated by the inhibition of Th1-cell differentiation and may increase the susceptibility to infectious complications after accidental or surgical injury.

## CLINICAL PERSPECTIVES

• Severely injured patients are highly susceptible to nosocomial infections due to the development of immunosuppression that is associated with an altered *ex vivo* T-cell responsiveness.
• We observed that in a murine model of traumatic skeletal muscle injury NK cells are recruited to the lymph node draining the site of injury and impair the development of protective Th1-cell responses. The NK cell migration is dependent on TLR4–MyD88 signalling and the presence of bystander T-cells.
• Breaking the recruitment of NK cells after injury might prevent the limitation of Th1-cell differentiation and, thus, might reduce the risk of nosocomial infections after severe injury.

## Online data

Supplementary data
