# Supplementary material for: Breaking the co-operation between bystander T-cells and natural killer cells prevents the development of immunosuppression after traumatic skeletal muscle injury in mice
Source: Clin Sci (Lond). 2015 Mar 19;128(Pt 11):825–38. doi: 10.1042/CS20140835 (PMC4557401; doi:10.1042/CS20140835)
Supplement: Supplementary data [file cs1280825ntsadd.pdf]

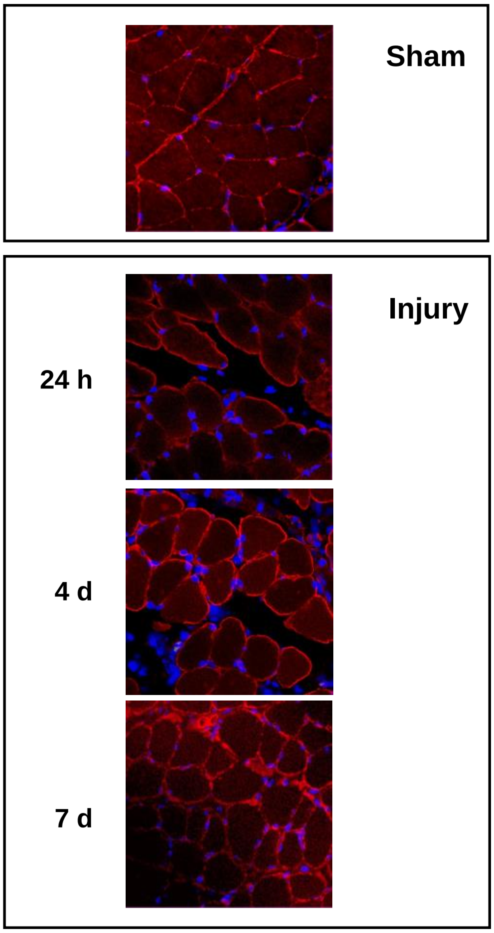

Figure S1 Mechanical injury disturbs muscle fiber organization
For histological analyses, the gastrocnemius muscles were prepared at different time points (24 h, 4 d, 7 d) after injury or sham treatment, frozen in pre-cooled isopentane, and embedded in Neg-50 (Richard-Allan Scientific, Kalamazoo, MI). Sections of 7 µm were fixed in 96% ethanol and air-dried. After blocking with 1% BSA/10% normal mouse IgG (BD Biosciences, Heidelberg, Germany) the sections were sequentially incubated with antibodies against Laminin-2 (α-2-chain, clone 4H8-2, 1:400; Alexis Biochemicals, Loerrach, Germany) and Cy3-labeled donkey anti rat IgG (1:200; Jackson ImmunoResearch, West Grove, PA). The sections were embedded in DAPI-containing Vectashield (Vector, Burlingame, CA). Images of representative sections were taken using the confocal laser scanning microscope Axiovert 100 M with a LSM 510 Laser-Scanning-Module. Magnification 400x. Ten sections per muscle were prepared and analyzed. One representative section is shown for each time point. The red color indicates laminin-2-expressing muscle cells. Nuclei are stained in blue.

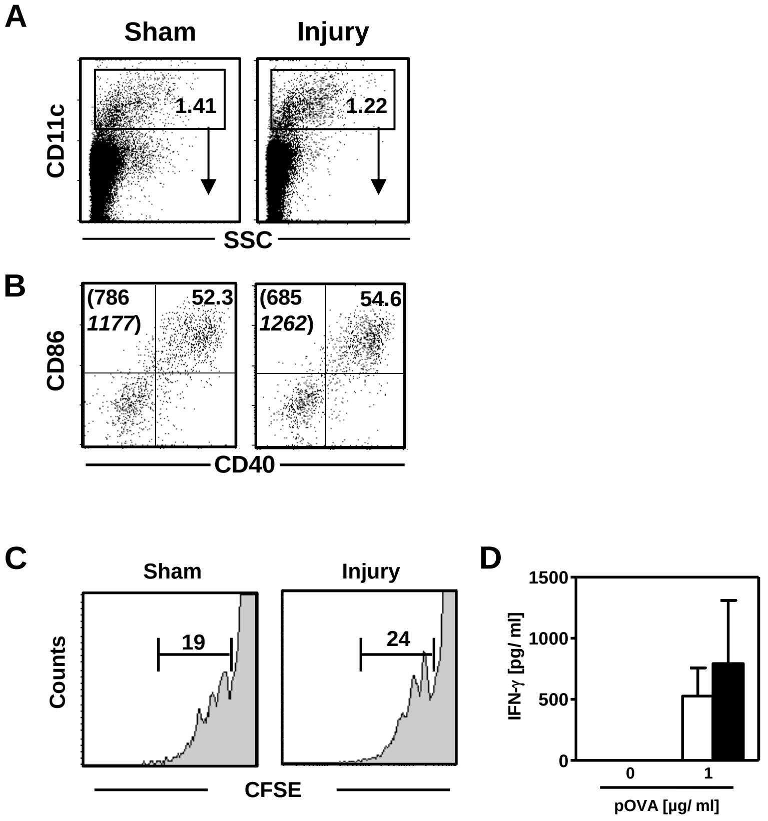

Figure S2 The function of antigen-presenting cells in popliteal lymph nodes is not disturbed after injury

Seven d after injury or sham treatment, popliteal lymph node (pLN) cells were pooled per group. (**A, B**) pLN cells were stained against CD11c, CD86, and CD40. (**A**) CD11c^{+} cells were gated (numbers indicate the percentage of CD11c^{+} cells in total pLN cells). (**B**) Numbers in the upper right quadrant of dot plots indicate the percentage of CD86^{+}CD40^{+} cells in gated CD11c^{+} cells. Numbers in the upper left quadrant indicate the MFI of CD86 (top) and CD40 (bottom) of CD40^{+}CD86^{+} cells. Dot plots are representative for 4 independent experiments with similar results. (**C, D**) Antigen-presenting cells (APCs) were prepared by depletion of CD3^{+} T-cells and were co-cultured with CFSE-labeled OVA-specific T-cells in the presence of ovalbumin peptide (pOVA) for 3 d. (**C**) The CFSE dilution in CD4^{+}KJ1-26^{+} OVA-specific Th-cells was determined by flow cytometry. Numbers in the histograms that are representative for 3 independent experiments indicate the percentage of OVA-specific T-cells that had undergone at least one cell division. (**D**) Content of IFN-γ in the supernatants. Data show mean+SD of triplicate cultures and are representative for 3 independent experiments with n=3 mice per group.

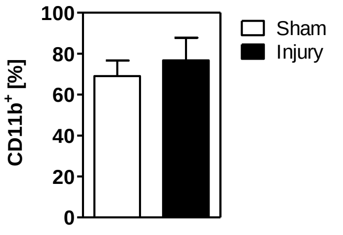

Figure S3 Myeloid cells in draining lymph nodes after injury in the absence of lymphocytes

Recombination-activating gene (RAG) $2^{-/-}$ mice underwent injury or sham treatment. Twenty-four h later single cell suspensions of popliteal lymph nodes were prepared and stained against CD11b. Cells were further analyzed by flow cytometry. Data show the mean+SD of the percentage of $CD11b^{+}$ cells in the lymph node of individual sham or injured mice (n=3 per group).

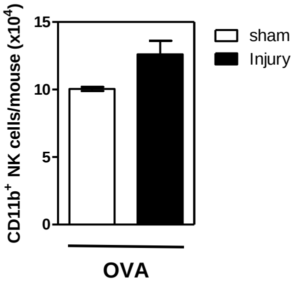

Figure S4 Recruitment of Natural Killer cells after application of ovalbumin Twenty-four hours after injury or sham treatment ovalbumin (OVA) was injected s.c. into both hind footpads. Popliteal lymph node cells were prepared 24 h later, pooled per group, and stained against CD3, DX5, and CD11b. The percentage of $CD3^-DX5^+CD11b^+$ Natural Killer (NK) cells was determined by flow cytometry and the absolute cell number per mouse was calculated. Data show mean+range of 2 experiments each with n=3 mice per group. For further details see the section Materials and Methods, Flow Cytometry.
